# Analysis of Accelerometer and GPS Data for Cattle Behaviour Identification and Anomalous Events Detection

**DOI:** 10.3390/e24030336

**Published:** 2022-02-26

**Authors:** Javier Cabezas, Roberto Yubero, Beatriz Visitación, Jorge Navarro-García, María Jesús Algar, Emilio L. Cano, Felipe Ortega

**Affiliations:** 1 Data Science Laboratory, University Rey Juan Carlos, 28933 Móstoles, Spain; roberto.yubero@urjc.es (R.Y.); beatriz.visitacion@urjc.es (B.V.); j.navarro.2016@alumnos.urjc.es (J.N.-G.); mariajesus.algar@urjc.es (M.J.A.); emilio.lopez@urjc.es (E.L.C.); felipe.ortega@urjc.es (F.O.); 2 Quantitative Methods and Socioeconomic Development Group, Institute for Regional Development, University of Castilla-La Mancha, 02071 Albacete, Spain

**Keywords:** animal behaviour, pattern recognition, anomaly detection, clustering, spectral analysis, accelerometer sensor, GPS sensor

## Abstract

In this paper, a method to classify behavioural patterns of cattle on farms is presented. Animals were equipped with low-cost 3-D accelerometers and GPS sensors, embedded in a commercial device attached to the neck. Accelerometer signals were sampled at 10 Hz, and data from each axis was independently processed to extract 108 features in the time and frequency domains. A total of 238 activity patterns, corresponding to four different classes (*grazing*, *ruminating*, *laying* and *steady standing*), with duration ranging from few seconds to several minutes, were recorded on video and matched to accelerometer raw data to train a random forest machine learning classifier. GPS location was sampled every 5 min, to reduce battery consumption, and analysed via the k-medoids unsupervised machine learning algorithm to track location and spatial scatter of herds. Results indicate good accuracy for classification from accelerometer records, with best accuracy (0.93) for *grazing*. The complementary application of both methods to monitor activities of interest, such as sustainable pasture consumption in small and mid-size farms, and to detect anomalous events is also explored. Results encourage replicating the experiment in other farms, to consolidate the proposed strategy.

## 1. Introduction

Monitoring activity of animals in livestock farms can provide relevant indicators about their health and welfare level. In fact, ensuring animal well-being through objective evidence has become a major concern for both cattle producers and consumers [1]. For example, EU Directive 98/58/EC regarding the protection of animals kept in farms [2] introduces general rules for protecting all animals species for production of food, wool, skin, fur or other farming purposes. Later on, EU legislation has been progressively extended to increase the well-being of farmed animals. Additionally, current EU regulation regarding organic farming rules encourages high standards for animal welfare, requiring farmers to meet specific behavioural needs of animals [3].

The development of systems to gather and analyse animal behaviour data can certainly help cattle producers to meet these high quality standards. In recent years, wireless sensor networks (WSN) and Internet of Things (IoT) technologies have paved the way for implementing monitoring systems on farms [4,5,6]. Various methods have been proposed for automated recording and identification of animal activity in this context. Sensors embedded in electronic devices attached on animals legs or using neckbands can record activity information, with great detail. Then, activity patterns of interest can be revealed through the analysis of these behavioural records.

A frequent case is the use of accelerometers to create motion logs by tracking movement in a 3-D coordinate system. These devices have been used to register movements of human users [7]. Moreover, it is possible to estimate the vertical component and magnitude of the horizontal component of the user’s motion, even in absence of precise information about the position and orientation of the device with respect to the body [8,9]. Many previous studies of livestock behavioural activity on farms use accelerometers to gather data describing animal movements [10,11,12,13,14,15]. Furthermore, certain studies also use low-cost GPS devices to register the location of animals, augmenting the information obtained from accelerometers [16].

Machine learning (ML) classification algorithms can be used to classify cattle activity patterns automatically, based on registers from accelerometer and GPS sensors [17,18]. Previous studies have documented accurate identification of cattle standing and walking behaviour through accelerometer data [11,19], along with precise estimation of the duration of standing behavioural patterns [20].

A comprehensive survey [21], comparing previous studies on ruminant behaviour prediction, indicates that most of them are focused on identifying a predefined set of activities. For instance, Smith et al. [22] differentiate among five possible classes (*grazing*, *walking*, *ruminating*, *resting* and *other activities*), whereas Riaboff et al. [23] distinguish up to 13 different behavioural patterns. Usually, the initial problem is broken down into a set of “one-vs-all” binary classification tasks. Hence, individual outputs from each classifier must be integrated, which leads to some practical challenges. For instance, it is desirable to follow a robust methodology for data acquisition and feature engineering that can be shared among different classifiers [21,24]. In the same way, another limitation of previous studies is the relatively narrow focus on specific cases of outlier detection techniques, such as lameness [25], oestrus periods [14] or parturition events [18,26].

Likewise, current research works exhibit a noticeable scarcity with respect to the early detection of specific cattle social interactions at group level, especially when they lead to anomalous situations involving potential economic impact on livestock farm operations.

Wolf and other predator attacks constitute a prominent example of such anomalies. When there exists a potential threat of attack, herds change their behavioural patterns to put on an alert. They could also stop grazing and ruminating, or even move away to a different location. The growing and perceptible concern among farm producers on this matter, and the pressing need to find sustainable trade-off solutions, that preserves both protected species, such as wolves in the northwest of Spain and other countries, and farmers’ rights to continue their normal operations and guarantee their animals well-being, constitute a challenging issue yet to be solved.

Disease transmission represents another good example. Early detection and subsequent application of proper corrective actions bring in an opportunity to avoid a severe impact on productivity. Unusual resting behaviours, abnormal stance and gaits, the absence of vertical or horizontal neck movements or the observation of too slow displacements can provide key signals of possible ongoing diseases.

Similarly, despite not representing an anomalous activity itself, the detection of an unbalanced use of pasture land can also help farmers to develop strategies aimed at a more rational consumption of natural resources, achieving better management and saving costs. In this regard, recurrent grazing habits and lack of displacement to alternative areas may render valuable information to farmers on pasture land usage. Along these lines, recent results shown in [27] suggest that the combination of movement records and GPS location data can improve detection of anomalous situations on farms.

In this paper, we present a method to classify cattle behaviour from accelerometer and GPS data, collected from collars attached to cows in two field experiments. Time and frequency-domain features are extracted from accelerometer data, to train a supervised ML classification model for cattle behaviour. GPS data is processed with an unsupervised clustering method to estimate the number of herds and their spatial scattering. This general method can be applied to a wide range of scenarios. Furthermore, new activities could be incorporated to the classifier, provided that customised training data describing the new patterns of interest are obtained, following the same preparation procedure. In addition, potential applications for tracking interesting or anomalous activities, such as unbalanced use of pasture land, disease transmission or predator attacks, are also explored.

The rest of the paper is organized as follows. Section 2 describes the equipment and experimental setup for this study, along with our proposed method to analyse animal behaviour records. Section 3 presents the main results from the two field studies to validate the suggested approach. In Section 4, we discuss the main implications that can be drawn from experimental results, as well as potential practical applications of the proposed method. Finally, Section 5 concludes and describes further research directions.

## 2. Materials and Methods

### 2.1. Farms and Animals

In our study, we focus on beef cattle located on two different commercial farms, located in the Spanish provinces of Avila and Segovia, respectively. Herds raised on these farms comprise widespread breeds, including *Fleckvieh* or *Salers*, among others, along with native Spanish breeds, such as the *Berrenda en Colorado* (brindle cow in red) or the *Avileña-negra Ibérica* (Iberian Avila’s black). Most of time, animals were kept on pasture and moved freely within the farm limits. Cows were mainly fed with pasture, although they also received hay and concentrate supplements. A random sample of 30 cows in both livestock farms were equipped with accelerometer and GPS devices (see Section 2.2, below). Selected cows are representative of the most prevalent breeds in cattle from both farms, namely, *Fleckvieh*, *Salers*, *Berrenda en Colorado* and cross-bred dairy specimens.

### 2.2. Device and Data Loggers

We explore the use of two different procedures to monitor and analyse animal behaviour:

- *Tracking movement*: Detailed movement registries are recorded through triaxial accelerometers attached to the neck. In this way, we can identify more different behavioural patterns than when the accelerometer is installed on the leg.
- *Tracking location*: Animal location is registered with GPS sensors that periodically transmit this information to a centralized server in a cloud computing infrastructure.

**Figure 1:**
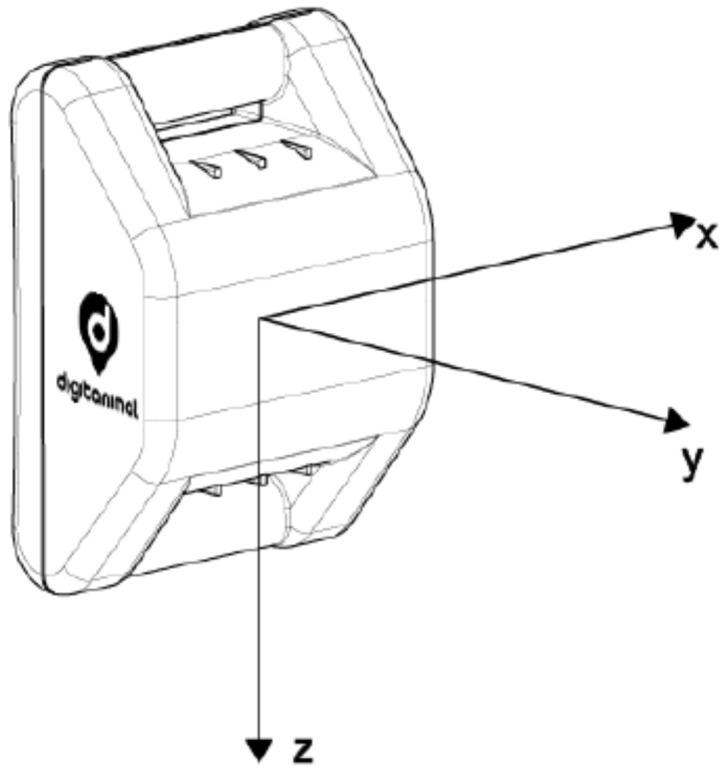
Monitoring device with 3-D accelerometer and GPS sensors. Coordinate axes represent movement directions tracked by the accelerometer.

**Figure 2:**
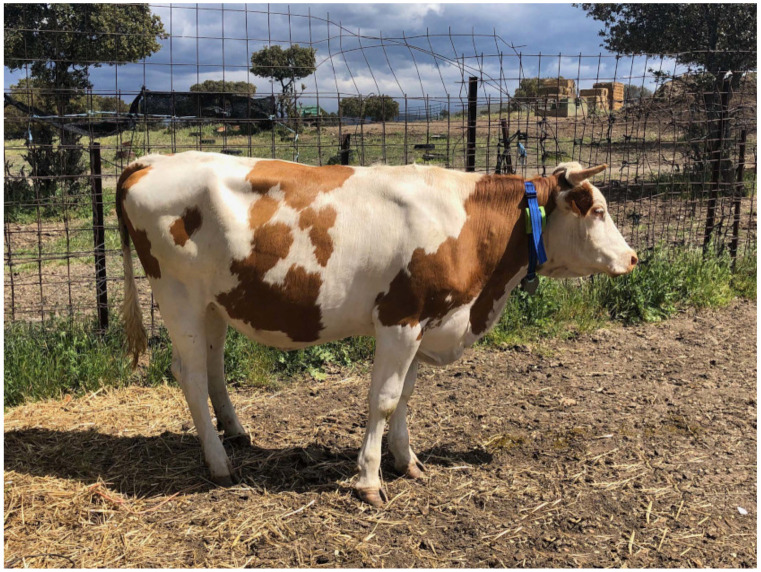
A Fleckvieh breed cow wearing the monitoring device, attached with a neckband.

Accelerometers and GPS sensors for this study were provided and installed by Digitanimal (https://digitanimal.com/?lang=en, accessed on 25 February 2022), a private company based in Madrid that develops innovative hardware and software animal monitoring solutions on farms. These sensors are integrated in an electronic device developed by this company, mounted inside a weatherproof plastic case and attached to the cow using a neckband. Figure 1 depicts a model for the collar case containing the device, and the three coordinate axes monitored by the accelerometer sensor. In turn, Figure 2 shows a cow of Fleckvieh breed wearing the neckband attaching the device to monitor movement and location.

Acceleration levels on cows necks are measured by using MEMS (Micro Electro Mechanical System) accelerometers. This type of accelerometer measures acceleration in 3 orthogonal directions (triaxial accelerometer, see Figure 1). The sensor captures DC (direct current or *offset*) acceleration (earth gravity), providing not only acceleration levels but also sensor orientation. It is a low-power consumption sensor, with a working temperature range of −40º to 85º Celsius, suitable for the required application. Raw data are acquired at a 10 Hz sampling frequency, using a dynamic range of ±2 g. Data are retrieved continuously since the sensor is connected and directly stored in plain text format in a SD memory card.

With respect to GPS sensors, they must send information at more widely spaced intervals than in the case of accelerometers in order to optimize battery consumption and, therefore, avoid premature battery draining. We must take into account that the monitor device is a commercial hardware solution, conceived to be affordable and remain operative over relatively long time periods (usually, 2–3 months). The GPS device is configured to use a maximum DOP (Dilution of Precision) threshold of 1, and to seek signal reception from a minimum of 7 different satellites. With this configuration, the estimated average measurement error is 1.7 m, and 90% of measurements present an error lower than 5.2 m.

A primary goal for this product is to avoid the need of frequent maintenance tasks (such as replacing the battery or the SD card), that would interfere with normal farm routines. In consequence, the sampling rate of GPS data is set to 5 min, that is, a single message is sent at the end of each 5-min interval. In spite of this initial specification, it is also possible that the GPS signal is lost in certain shadow regions on the farm, or that transmitted data do not successfully arrive at the server, due to propagation issues, network problems or other causes. For this reason, the system must be prepared to deal with missing data in location records.

Next, we describe the procedure for data acquisition, the proposed method for processing accelerometer and GPS data, as well as the approach for identification of animal behaviours using ML algorithms.

### 2.3. Accelerometer and GPS Data Collection

**Figure 3:**
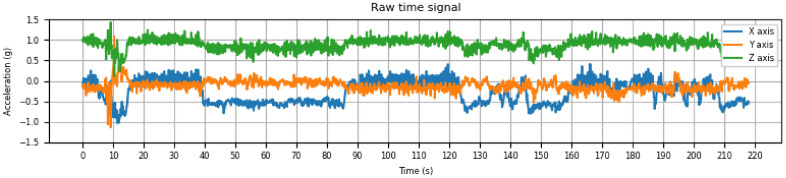
Raw signals recorded by the 3-D accelerometer for each coordinate axis.

Figure 3 shows an example of 3 raw signals produced by one of the accelerometers for 220 s. Each individual signal is the result of monitoring acceleration changes along a single axis. The blue signal corresponds to the X-axis, the orange signal represents oscillations along the Y-axis and the green signal stands for acceleration changes along the Z-axis. Raw signals recorded by each accelerometer are inputs for the feature extraction step in data analysis (see Section 2.6 for further details).

While triaxial accelerometers in collars store signal data locally, the GPS sensor monitors the location of the animal and periodically transfer these data to a central server each 5-min interval, containing the following attributes:

- id: Unique identifier for the monitored cow.
- timestamp: A timestamp value in the format YYYY-MM-DD HH:MM:SS.
- longitude: Longitude coordinate for the current animal position.
- latitude: Latitude coordinate for the current animal position.

### 2.4. Behavioural Observations

One of the main limitations to validate the automated detection of behavioural patterns in ruminants is the lack of a validation database, providing examples of specific behaviours and their associated patterns captured by the accelerometer. In our experiments, this is addressed by taking video recordings of a sample of animals on pasture fields, wearing the monitoring devices described above. Hence, the main goal of these recordings is to match each logged signal with its corresponding recorded behaviour. Furthermore, video recordings also allow double-checking the correct alignment between signals and video timestamps, a problem addressed via a specific methodology described in Section 2.5.

A team of 10 scientists were trained to supervise the recorded scene, annotating the timestamp and observed behaviours. Each scientist was responsible for tracking a single animal, annotating behavioural patterns over a 5 h session. Operators encoded activities on log files using a shared predefined nomenclature, described in the ethogram shown in Table 1. Annotated behaviours include: *grazing*, *ruminating*, *steady standing*, *laying* and *others*. The last category encompasses less frequent behaviours, such as running, scratching, drinking, calf nursing, etc.). The duration of individual behaviours was quite variable, ranging from few seconds (e.g., for *scratching*) to several minutes, in some cases (e.g., ∼16 min for a single instance of *ruminating* or ∼11.5 min for one instance of *laying*). Some behaviours required immediate reactions from human observers, therefore reducing the length of video recordings to identify them (e.g., in *running* operators must relocate to follow the animals).

Video recordings and observational log files derived from them were subsequently reviewed by independent supervisors, to ensure consistency of activity labelling between files. After an initial screening, 3 animals were selected for this analysis, as their activity logs provided the most accurate registries. A total of 238 unique behavioural patterns were identified from these recordings. These patterns, together with their associated signals recorded by accelerometers, constitute the gold standard for this study.

**Figure 4:**
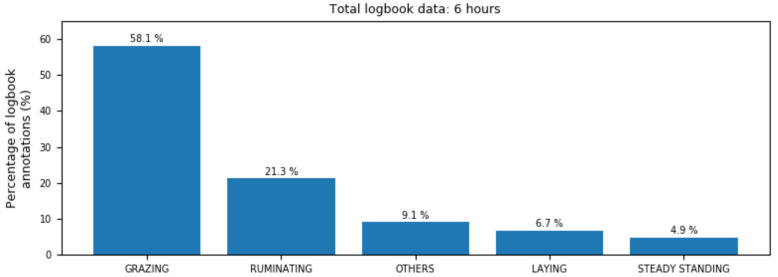
Proportion of observed behaviours of cows on the field, annotated by scientists.

Figure 4 reports the percentage of samples corresponding to each individual activity, over the total number of logged behaviours. Despite other activities were also annotated by scientists in observational logs, only the most frequent ones are considered in this classification analysis.

### 2.5. Alignment between Accelerometer Data and Observations

To facilitate the matching of signal and video records, a special procedure was followed to create a distinctive signature that clearly marked the start and the end of the experimental scope:

- Before the sensor collar is installed, the operator swings the collar for 1 min, so that a unique oscillation pattern is produced by the accelerometer on the 3 axes.
- When the experiment is finished and just after the collar is taken off from the animal, the operator swings again the collar for 1 min to reproduce the same unique pattern as in the starting point.

This pattern marking the start and the end of the experimental observation time cannot be reproduced naturally by cows while wearing the collar. Hence, this signature signal can be employed to fine tune the alignment between internal clocks in video cameras and the accelerometer clock.

### 2.6. Processing Accelerometer Data

**Figure 5:**
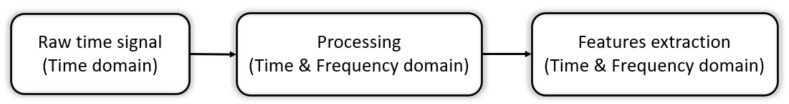
Overview of the proposed procedure for accelerometer data processing.

The procedure for accelerometer raw data processing consists of different steps, which are depicted in Figure 5.

**Figure 6:**
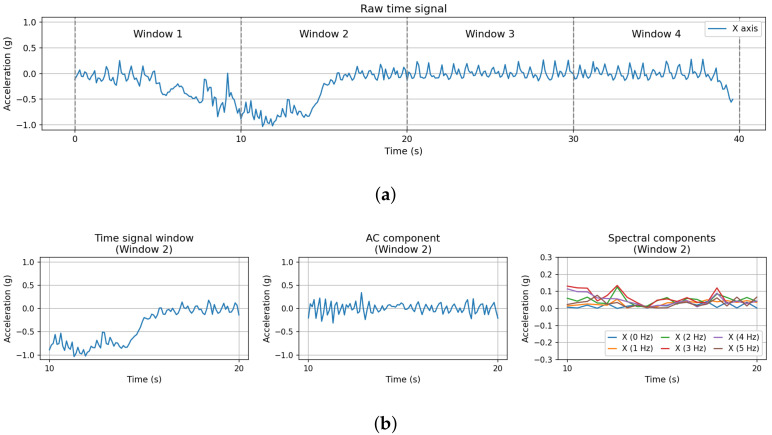
Time windows extracted from original signal generated by the accelerometer and their corresponding components. (**a**) Raw time signal divided in 4 windows. (**b**) Time-domain signal and components extracted from *Window 2* in Figure 6a.

In the first step, time signals are divided in 10 s consecutive, non-overlapping *time intervals* or *time windows*. As a result, each interval contains 100 consecutive samples, since 10 samples per second are generated using a 10 Hz sampling rate. From now on, we refer to the time intervals obtained from this process as $x_{i}\left(t\right)$, where *i* denotes the interval index. Figure 6a illustrates the result from this step, dividing the original signal (in this case, for the X-axis) in 4 different time windows, spanning adjacent intervals of 10 s. It must be remarked that, unlike many previous studies of this kind of data (see [21] for a comprehensive survey), we process the signals from each of the 3 accelerometer axes (X, Y, Z) separately. Previous studies analysing animal behaviour in wild habitats [28,29,30] suggest that this alternative data processing method can provide advantages for accurate detection of behavioural patterns, especially dynamic ones.

The second step in Figure 5 involves processing the time interval to obtain their AC (alternating current) component, along with its representation in the frequency domain, which are illustrated in Figure 6b. In this study, the AC component is extracted using a method that differs from several previous research works, where digital filters are applied to remove high-frequency noise and eliminate the DC component [31]. Hämäläinen et al. [9] show that problems may arise when the orientation of the sensor changes (e.g., due to sudden shakes) during data acquisition. Thus, they propose a simple alternative method to avoid these problems, calculating instead the “jerk” (acceleration change) between two consecutive samples. This approach renders orientation-independent features, avoiding the need to estimate the actual acceleration accurately.

**Figure 7:**
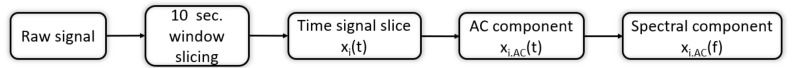
Pipeline performed within the accelerometer signal processing stage.

As a result, the AC component, identified as $x_{i-AC}\left(t\right)$, is computed as the regular difference between two consecutive time windows. Finally, the frequency-domain representation of the AC component is calculated in the last stage of this pipeline, as the basis for subsequent spectral processing. To achieve this, we compute the Fast Fourier Transform (FFT) [32,33] of the AC component, using a 1-s window size, a Hanning window type [34] and 50% overlapping between consecutive time windows. Figure 7 summarizes the pipeline for processing the raw signal from the accelerometer in the time and frequency domains.

Calculating the FFT of the AC component renders a spectrogram in a frequency range from 0 Hz to 5 Hz (according to Shannon’s theorem), with a 1 Hz resolution. For this purpose, the Python SciPy signal processing toolbox (https://docs.scipy.org/doc/, accessed on 25 February 2022) is used. This will be denoted as the *spectral component*, identified as $X_{i-AC}\left(f\right)$. The complete spectral component comprises 6 individual frequency components, corresponding to each of the 1-Hz resolution bands. The panels in the lower part of Figure 6 represent the three elements calculated in this second step. The lower-left panel represents slice 2 from the original signal in the top panel. The AC component extracted from the original signal in time interval 2 is shown in the lower-centre panel. Finally, the time-domain representation of the spectral component for each frequency band is depicted in the lower-right panel.

The three elements obtained from the preprocessing step (time window, AC component and spectral component) are inputs for the extraction of different features, performed in the third step of Figure 5. Finally, these features are used for automated behaviour detection with ML algorithms. When the data inputs are the time window and the AC component, features obtained in this way correspond to the time domain. In turn, when the input is the spectral component, resulting features correspond to the spectral domain. Table 2 describes the list of features extracted from each data input. Details about their computation are explained below.

#### 2.6.1. Time Domain Features

Table 2 shows the features obtained for each type of data input from accelerometer signals. Using the raw accelerometer axis (X, Y, Z) input, the following features are obtained (see first row in Table 2): mean, maximum, minimum, 5th percentile and 95th percentile. A total of 15 time features are extracted, 5 features per each accelerometer axis (X, Y, Z). Likewise, using the AC component representation in the time domain as a data input (see second row in Table 2) the mean, maximum, standard deviation (STD), skewness, kurtosis, 5th percentile and 95th percentile features are computed. A total of 21 features are extracted using this data source, that is, 7 features per each accelerometer axis.

#### 2.6.2. Frequency Domain Features

The spectrogram represents how acceleration levels progress for each frequency and time instant. According to the processing parameters previously defined, the spectrogram of the AC component comprises 6 different frequency bands, at 0 Hz, 1 Hz, 2 Hz, 3 Hz, 4 Hz and 5 Hz. Since the spectrogram represents information in 3 dimensions simultaneously (time, frequency and amplitude), it cannot be used directly for feature extraction. To achieve this, the spectrogram is decomposed of frequency, obtaining 6 spectral series that progress along time. Figure 6b shows the resulting spectral series for a certain spectrogram.

Once these spectral series are computed for the AC component, they can be used for feature extraction. The third row of Table 2 presents the features that are extracted for each spectral series: root mean square (RMS) value, standard deviation (STD), minimum and maximum value. A total of 72 spectral features are obtained, that is, 4 features for 6 spectral series, resulting in 24 features per axis.

### 2.7. Processing GPS Data

Location records transferred by GPS sensors to the server are stored in CSV files. There is one file for each tracking collar attached to a cow. To start off, data in all CSV files are coalesced into a single file and records are ordered according to their timestamp value. Duplicate entries that might have been incorrectly recorded or transferred are also elided in this first step. After this, we must clean the dataset filtering incorrect location entries. This may be caused, for instance, due to inaccurate location detection by the sensor in areas of the farm where GPS coverage is insufficient. To attain this, the daily average values for latitude and longitude coordinates are calculated for each monitored farm. Then, any location registry further than 1 km from the average position is eliminated. This figure is well above the average value of the overall area of both livestock farms under analysis (about 50 hectares, in both cases), to filter out clear data registration errors. Once GPS data are completely prepared, the relevant attributes (id, timestamp, longitude and latitude) can be used.

### 2.8. Machine Learning Algorithms

Features extracted from accelerometer signals are used to train a supervised ML algorithm for behavioural pattern classification, whereas GPS location data is analysed through an unsupervised machine learning method, to detect anomalous activity patterns. Details on these analyses using machine learning models are provided below.

#### 2.8.1. Behaviour Classification Based on Accelerometer Data

Classification of behavioural data from field experiments is performed using the random forests (RF) algorithm [35]. This tool has been selected due to the high number of descriptive features available and the capacity of RF to automatically identify important features to detect each individual behaviour.

The complete set of 238 behavioural samples, including the 108 features extracted from accelerometer signal processing, is split into 5 different folds, following a stratified random sampling approach [36]. Then, a multi-class RF classification algorithm is trained for every fold using 75% of data and the remaining 25% for testing. Video recordings are combined with these input data to produce a validation database. The target categories for the classification task are *grazing*, *ruminating*, *laying* and *steady standing*, while patterns included in category *others* are filtered out, since not enough samples for each individual behaviour in this group are available to identify them accurately. The hyperparameters selected for RF are the following: we use information gain (*entropy*) to measure the quality of splits; the minimum number of samples required to split an internal node is set to 20; we select using out-of-bag samples to estimate the generalization score and we build 100 trees for each forest. Then, the importance of each feature to identify individual activities is obtained in every trained model. Finally, all feature importance values per activity are averaged over the 5 folds to report the final results.

#### 2.8.2. Automated Detection of Herd Scattering Using GPS

The main objective in our analysis of GPS location data is to automatically identify groups of monitored cows within the limits of the farm and sudden changes in the scattering of a given group. Rapid modifications in animal dispersion within a certain group may indicate the occurrence of anomalous events that must be reported to farm operators and managers.

In the first place, data for each livestock farm is identified and analysed separately. Given a location dataset describing the situation in a farm, a centre location for every group of animals must be identified. Then, the dispersion of animals around their corresponding group centre must be estimated and tracked, to account for abrupt alterations. We use the *Euclidean* distance ($L_{2}$ norm) [37] to measure the separation between any two cows, and generate the distance matrix for all animals in the farm.

Identifying the groups and their representative location leads to an unsupervised learning task. Among the different alternative algorithms that can be applied, partitioning clustering algorithms [38,39] provide a convenient solution, as the total number of location points in each farm is not large. Although the k-means algorithm [37] is a popular solution for this kind of problems, we found that, in many cases, it does not provide representative locations for each group of animals in this application. The main cause behind this problem is the frequent presence of outliers in animal groups, that is, cows that are well-separated from the rest of members of the same cluster, thus pulling the location of the k-means centre for that group.

Due to this, a more robust clustering algorithm, insensitive to the presence of outliers in a cluster, must be employed. The k-medoids algorithm [38,39] forces the selection of one of the actual location points in a certain cluster to act as the centre for that group. We found that cluster identification following this approach is much more reliable and better matches extant information from farm workers and managers about the number and location of herds. The appropriate number of clusters for each farm is selected by calculating the within-clusters sum of squares (WCSS) for different values of *k*, evaluating the cohesion of clusters in each case. Then, a scree plot of WCSS against *k* is generated and we choose the value for *k* using the elbow method [37,40]. Alternatively, farm managers could override this choice of *k* by entering extant information about the estimated number of herds.

**Figure 8:**
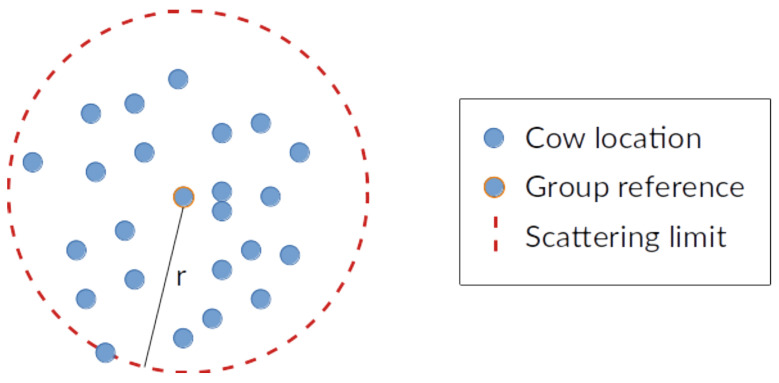
Detection of the scattering limits for a herd. The farthest animal assigned to that group determines the scattering radius *r*.

Once the number of herds and a reference location for each group are found, we turn to the problem of estimating the scattering of animals in a given group from their reference point. In this case, we opt for choosing the farthest animal assigned to the cluster as the delimiter of the maximum scattering range for that group, as shown in Figure 8. Since we use the Euclidean distance to measure proximity between cows, we effectively establish a circular region of radius *r* equal to the distance from the reference location in the group to the farthest member of that herd.

This procedure is periodically repeated for every new sample of locations sent by GPS sensors from the farm. For each new sample, the total number of groups, the reference location and the estimated value of *r* for each group are computed and stored.

## 3. Results

In this section, we summarize the results from the field experiments to identify animal behavioural patterns using the features extracted from accelerometer signals represented in the time domain and the frequency domain, and GPS location data.

### 3.1. Relevant Classification Features

Table 3 shows the rank and feature importance values [35,41] (mean accumulation of impurity decrease within each tree, known as Mean Decrease in Impurity or MDI), averaged from the five RF models trained with time domain and frequency domain features obtained from accelerometer signals. For the sake of conciseness, here we only report the top-five features identified for each activity. Graphs displaying the complete set of features for each activity and their associated importance values are presented in Appendix A.

We can spot several interesting traits regarding the most important features used by the RF algorithm to identify each behaviour. In the case of activity *grazing*, the most important features to detect this pattern are related to movement along the Z-axis. This is consistent with the observed movements, involving vertical necks displacements as the cow lows down its head to eat pasture and raise it up to continue chewing. Moreover, we also notice that two out of the top five features come from the frequency domain representation of the AC component. This confirms the usefulness of the spectral analysis of accelerometer signals for animal behaviour recognition. Another salient example of the key role of spectral components in activity detection is the case of *steady standing*. four out of the top five features come from the AC component processing in the frequency domain.

### 3.2. Classification Performance Metrics

Table 4 presents several performance metrics computed for the RF classification model, namely, accuracy, recall and AUC [42,43]. In general, classification accuracy attained by this algorithm was good for all behavioural patterns, with the highest score for *grazing* and the lowest for *ruminating*. However, recall metrics drop for activities with fewer samples in the dataset, such as *laying* or *steady standing*. Since we are developing a general detection procedure, that targets a variety of activities, the algorithm still presents limitations detecting all instances from under-represented categories, with fewer samples in the dataset.

## 4. Discussion

Previous research has shown the high interest of animal behaviour identification on farms [4,5,6]. Therefore, this work aims to propose a general procedure to recognize multiple activities based on accelerometer and GPS data. On top of this, previous studies has been restricted, so far, to the use of one of these two types of data sources for tracking animal behaviour, with only recent exceptions [16,18,44]. In this work, we explore the potential of combining data from both types of sensors to achieve a more advanced activity pattern identification.

### 4.1. Classification Model from Accelerometer Data

As described in Section 2.6, a separate analysis of accelerometer signals over each axis (X, Y, Z) along with the use of jerk filters and spectrograms to compute relevant features is proposed. Previous studies have shown [30] that the combination of this data processing method with classification trees ML algorithms (like the RF ensemble learning method applied in this work) can render good results for identification of animal behavioural patterns.

According to the feature importance metrics reported by the assessment of the RF classification model, shown in Table 3, time-domain features play an important role in the classification of certain behaviours such as *laying* or *ruminating*, where animals tend to remain relatively still. In our data processing method, this is linked to the absence of sudden shakes (“jerk” or “AC component” in this study), which turn the AC signal quite stable over time. In turn, frequency-domain features are also relevant for detection of dynamic behaviours such as *grazing*, or patterns with sudden activity peaks in any axis such as *steady standing*, better captured by our definition of AC component.

In this regard, it is of key importance that the internal clock used by the accelerometer marks precise regular intervals between samples. Otherwise, digital signal processing techniques to obtain the spectrogram of the AC component for different frequency bands will not be applicable, in case that sampling intervals present irregularities. However, results from this field study with commercial, low-cost equipment are limited by the accuracy of captured signals (that can be subject to sensor failures, battery drain due to climatic conditions and other adverse situations) and the ability to precisely correlate behaviours observed by human operators and registered on video recordings with the corresponding patterns captured by sensor devices. For example, as shown in Section 3.2, *grazing* was the most frequent activity pattern detected, which is in line with results from previous studies [16]. Possibly due to this high number of available samples identification of most frequent behaviours is more accurate than for other less represented patterns, according to performance metrics in Table 4.

On top of this, 9.1% of behaviours included in the study were labelled as *other*. However, detailed annotations were taken by operators regarding actions jointly accounted for in the omnibus *other* category. These include, among others, cows feeding younger calves, running cows or animals licking themselves. Some of these behaviours were correctly logged by human operators but not enough signal samples were obtained to generalize their detection to other cases. As a result, this study confirms that the proposed methodology could be generalized to other behavioural patterns, as soon as new data becomes available. An important implication in this sense is the absence of publicly available online reference datasets, registering data captured by sensors and their related activity patterns. Therefore, addressing this lack of validation databases could be a very useful contribution in further research works.

### 4.2. Potential of GPS Data for Activity Detection Based on Herd Scattering

**Figure 9:**
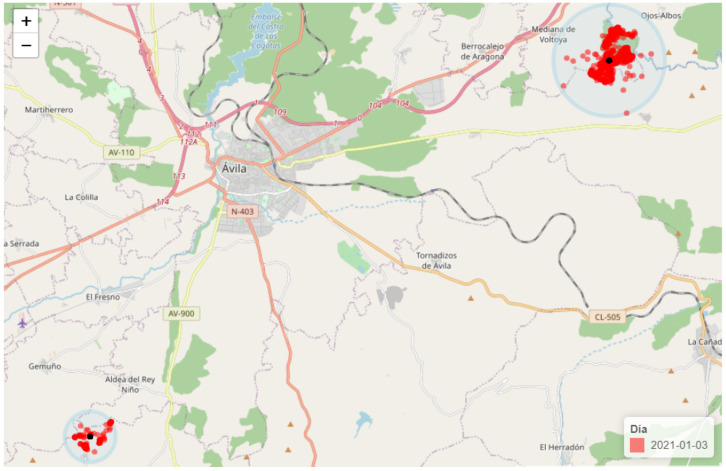
Example of automated detection of location and scattering of two different herds in one of the farms, near Ávila (Spain).

Figure 9 presents the result of a preliminary algorithm for automated detection of herds and within-herd spread, based on GPS data, corresponding to the livestock farm in Avila. The red dots depict the location of animals tracked by the GPS sensors. The map shows two separate herds, represented by the algorithm via the identified k-medoids for each group (black point). Then, the algorithm calculates the scattering of animals around the cow selected as the representative centre for that group. The algorithm could also detect changes in the radii calculated for each herd, following a basic procedure based on change point detection [45].

As shown in previous research [27], accelerometer and GPS data can be combined to detect anomalous events, such as unbalanced use of pasture land or disease transmission, among others. Table 5 describe some potential cases in which both components could be combined to eventually provide farmers with the proper tool for an early detection.

In the case of predator attacks, cows are vigilant and in state of alert. This natural response to a feasible external threat translates into the detection of noticeable periods of time in which cows are not moving their heads (grazing and ruminating activities are stopped). Likewise, herds may move away to an alternative location quite rapidly to mitigate the detected risk [46,47].

As for the use of pasture land, accelerometer data obtained from monitored cows would inform about grazing activities. For its part, GPS data would provide clear indication of the areas on which such activity occurs. Despite not being an anomalous activity, this information may assist farmers to better manage resources and costs, or even reduce pasture land required which is considered as a top priority demand [16,48,49].

Finally, the lack of vertical or horizontal movements in cow necks, an abnormal stance and gait, an unusual resting behaviour or too slow (or even non-existent) displacements detected via GPS data could offer an complementary perspective to detect disease transmission, whose modelling process would also require health scoring for each monitored animal. Early disease detection could prevent severe cases and facilitate immediate application of treatment measures, reducing productivity loss [50,51].

This procedure is also compatible with the automated detection of the number of herds within the farm limits or with manual configuration of the number of herds to be tracked, introduced by human users. Additionally, the proposed method can also be integrated in existing tools for animal monitoring on farms. Operators can configure the appropriate parameters to raise notifications, based on their own management experience with animals. As additional data are tagged and become available, the tool can be linked to the detection of particular patterns of interest (predator attacks, parturition, etc.). Again, the absence of publicly available datasets that can serve as a benchmark for this type of automated tools in animal behaviour recognition calls for filling this gap in further research.

Tracking the evolution of these indicators over time, it would be possible to identify two types of interesting changes:

- As herds move around the terrain, the reference animal representing that herd will register such displacement. Therefore, at the end of the day farm operators and managers can review the trajectory followed by different herds, leading to a more precise estimation of pasture consumption.
- Changes in the scattering radius *r* calculated for each herd may indicate interesting behavioural patterns happening to that group of animals. In particular, a sudden increase in the value of *r* may indicate among other possibilities) the attack of potential predators or other threats.

Another interesting line for further research is exploring the formal combination of activity records from accelerometers and GPS, for instance, through information fusion techniques [44]. The validity of this approach has already been tested for the case of outlier detection. Moreover, the only previous work that combines GPS and accelerometer datasets [16] is tailored to detecting a single behaviour (grazing) and just employ the GPS coordinates to locate every behaviour interval. However, there is a clear potential in the simultaneous utilization of features extracted from both accelerometer and GPS location data analysis to improve the recognition of animal activity patterns on farms.

## 5. Conclusions

In this work, we present a new method for automated classification of animal behavioural patterns, through the analysis of activity data registered by a triaxial accelerometer and a GPS sensor. A unique aspect introduced in this approach is the application of techniques for spectral analysis of accelerometer signals in the frequency domain. Descriptive features derived from the spectrogram of these signals play an important role in detecting certain patterns of interest, such as grazing (the most frequent activity observed) or steady standing. Likewise, this method is not restricted to a particular behavioural pattern and it can be readily generalized to any behaviour of interest, provided that labelled activity data is available. Furthermore, the analysis of GPS data recording animals locations through unsupervised machine learning algorithms enables the detection of groups of animals and their dispersion, which can be regularly tracked and reported to users. Jointly, results from these two analyses can build a more complete picture of activity logs and facilitate decision-makers the necessary information to oversee pasture consumption, develop actions in response to anomalous events and improve animal welfare in their farms.

## Figures and Tables

**Table 1:** Behavioural ethogram describing frequent activities observed by operators in the experiment, ordered by total duration of recorded video evidence.

Behaviour	Code	Total Durat. (sec.)	Description
Grazing	GRA	12,056	Regularly lowering and raising its head to eat pasture, while standing or walking slowly
Ruminating	RUM	4429	Ruminating previously eaten food, while standing or laying
Laying	LAY	1940	Laying on the ground without performing any other relevant activity
Steady standing	STA	1011	Standing almost still without performing any other relevant activity
Walking	OTH#WALK	509	Walking at normal pace with calm steps
Licking	OTH#LICK	414	Noticeably turning its neck to lick itself
Scratching	OTH#SCRA	159	Raising one leg to scratch its head or body (also specified if scratching against a tree)
Running	OTH#RUN	94	Moving at high pace with quick steps
Drinking	OTH#DRI	93	Lowering its head to drink water
Calf nursing	OTH#NUR	30	Steady while nursing a calf

**Table 2:** Features extracted from each input generated after preprocessing the accelerometer signal.

Data Input	Feature	Description
Raw accelerometer axis (X,Y,Z)	Mean	Average value of signal
	Max	Maximum value of signal
	Min	Minimum value of signal
	Q5	5th percentile of signal values
	Q95	95th percentile of signal values
AC component (time domain) $x_{i-AC}\left(t\right)$	Mean	Average value
	STD	Standard deviation of values distribution
	Kurtosis	Kurtosis of values distribution
	Skewness	Skewness of values distribution
	Max	Maximum value
	Q5	5th percentile of values
	Q95	95th percentile of values
AC component (freq. domain) $x_{i-AC}\left(f\right)$	RMS	Root mean square spectral density
	STD	Standard deviation spectral density
	Min	Minimum value spectral density
	Max	Maximum value spectral density

**Table 3:** Identified animal behaviours, top-5 features used by trained RF models to classify them and their importance (MDI), averaged over the 5 RF models.

Behaviour	Rank	Feature	Avg. MDI
Grazing	1	Z_AC_Q5	0.06798
	2	Z_AC_STD	0.06274
	3	Z_2Hz_RMS	0.06189
	4	Z_AC_Q95	0.06115
	5	Z_1Hz_RMS	0.06007
Laying	1	Y_Q95	0.06129
	2	Z_AC_Q5	0.05319
	3	Y_MAX	0.04246
	4	Y_AC_Q95	0.03975
	5	Y_MEAN	0.03601
Ruminating	1	Z_AC_Q5	0.06787
	2	Z_AC_Q95	0.04863
	3	X_Q5	0.04849
	4	Z_AC_STD	0.03537
	5	Y_Q_95	0.03486
Steady standing	1	X_1Hz_MIN	0.06075
	2	X_5Hz_MIN	0.04870
	3	X_3Hz_MIN	0.04429
	4	X_2Hz_MIN	0.04194
	5	X_AC_KURT	0.04022

**Table 4:** Performance metrics for the RF classification model. All metrics are average values over the 5 folds.

Behaviour	Accuracy	Recall	AUC
Grazing	0.93	0.945	0.974
Laying	0.907	0.611	0.894
Ruminating	0.881	0.893	0.967
Steady standing	0.922	0.58	0.912

**Table 5:** Examples of anomalous/interesting activities and how analysis of accelerometer and GPS data can be applied to detect them.

Activity of Interest	Accelerometer Data	GPS Data
Predator attacks	Vertical axis with no movement	Quick displacement to alternative location; possible successive relocations
Pasture land use	Detection of grazing behaviour	Mapping of areas under use (presence longer than a certain time threshold)
Disease transmission	Detection of steady-standing or laying behaviours	Erratic movements; very slow transitions to alternative areas

**Figure A1:**
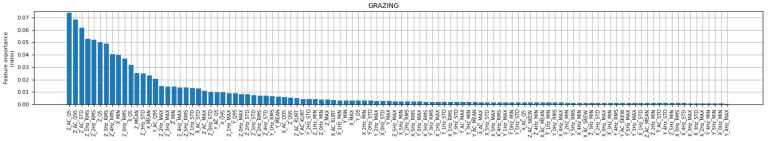
Feature importance in detection of grazing behaviour.

**Figure A2:**
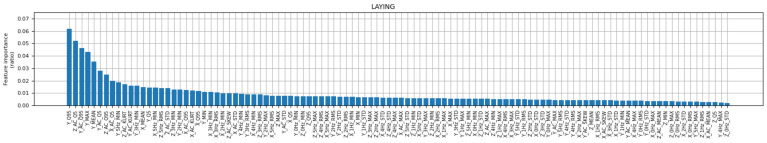
Feature importance in detection of lying behaviour.

**Figure A3:**
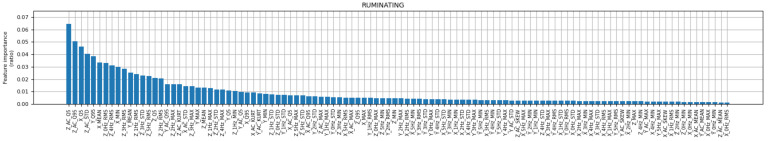
Feature importance in detection of ruminating behaviour.

**Figure A4:**
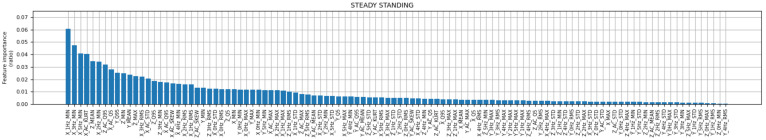
Feature importance in detection of steady standing behaviour.

## Abbreviations

The following abbreviations are used in this manuscript:

AC	Alternating Current
AUC	Area Under the Curve
CSV	Comma Separated Values
DC	Direct Current
FFT	Fast Fourier Transform
GPS	Global Positioning System
IoT	Internet of Things
MDI	Mean Decrease in Impurity
MEMS	Micro Electro Mechanical System
ML	Machine Learning
PCA	Principal Components Analysis
RF	Random Forests
RMS	Root Mean Square
ROC	Receiver Operating Characteristic
SD	Secure Digital
STD	Standard Deviation
WCSS	Within-Cluster Sum of Squares

## Appendix A

Next, we provide several graphs presenting the importance values of each feature extracted from the analysis of signals captured by triaxial accelerometers, for identification of the main behavioural patterns considered in this study, as reported by the RF algorithm.
